# Oxidative stress in intervertebral disc degeneration: Molecular mechanisms, pathogenesis and treatment

**DOI:** 10.1111/cpr.13448

**Published:** 2023-03-13

**Authors:** Yidian Wang, Huiguang Cheng, Tao Wang, Kun Zhang, Yumin Zhang, Xin Kang

**Affiliations:** ^1^ Department of Joint Surgery, Honghui Hospital Xi'an Jiaotong University Xi'an Shaanxi China

## Abstract

Low back pain (LBP) is a leading cause of labour loss and disability worldwide, and it also imposes a severe economic burden on patients and society. Among symptomatic LBP, approximately 40% is caused by intervertebral disc degeneration (IDD). IDD is the pathological basis of many spinal degenerative diseases such as disc herniation and spinal stenosis. Currently, the therapeutic approaches for IDD mainly include conservative treatment and surgical treatment, neither of which can solve the problem from the root by terminating the degenerative process of the intervertebral disc (IVD). Therefore, further exploring the pathogenic mechanisms of IDD and adopting targeted therapeutic strategies is one of the current research hotspots. Among the complex pathophysiological processes and pathogenic mechanisms of IDD, oxidative stress is considered as the main pathogenic factor. The delicate balance between reactive oxygen species (ROS) and antioxidants is essential for maintaining the normal function and survival of IVD cells. Excessive ROS levels can cause damage to macromolecules such as nucleic acids, lipids, and proteins of cells, affect normal cellular activities and functions, and ultimately lead to cell senescence or death. This review discusses the potential role of oxidative stress in IDD to further understand the pathophysiological processes and pathogenic mechanisms of IDD and provides potential therapeutic strategies for the treatment of IDD.

## Graphical abstract

Mitochondria are a major site of intracellular reactive oxygen species (ROS) production. Under physiological conditions, 0.2%–2% of electrons in the mitochondrial electron transport chain (ETC) leak out of the ETC and interact with oxygen to produce superoxide and H_2_O_2_. In addition, NADPH oxidase (NOX4) is up‐regulated in senescent cells, which can increase the production of ROS. In degenerative IVD, the levels of cell senescence and mitochondrial dysfunction increase, which contribute to the abnormal increase of intracellular ROS level. At the same time, ROS is also an important factor leading to cell senescence and mitochondrial dysfunction, which forms a positive feedback loop. Disturbance of redox status in degenerative IVD manifests as increased ROS production on the one hand, and decreased expression levels and activities of antioxidant substances on the other hand. Therefore, the imbalance of ROS production and scavenging in degenerative IVD leads to the disturbance of redox status, which causes oxidative damage to intracellular biomacromolecules and subsequent cellular dysfunction.

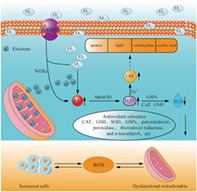

## 1 INTRODUCTION

**FIGURE 1:**
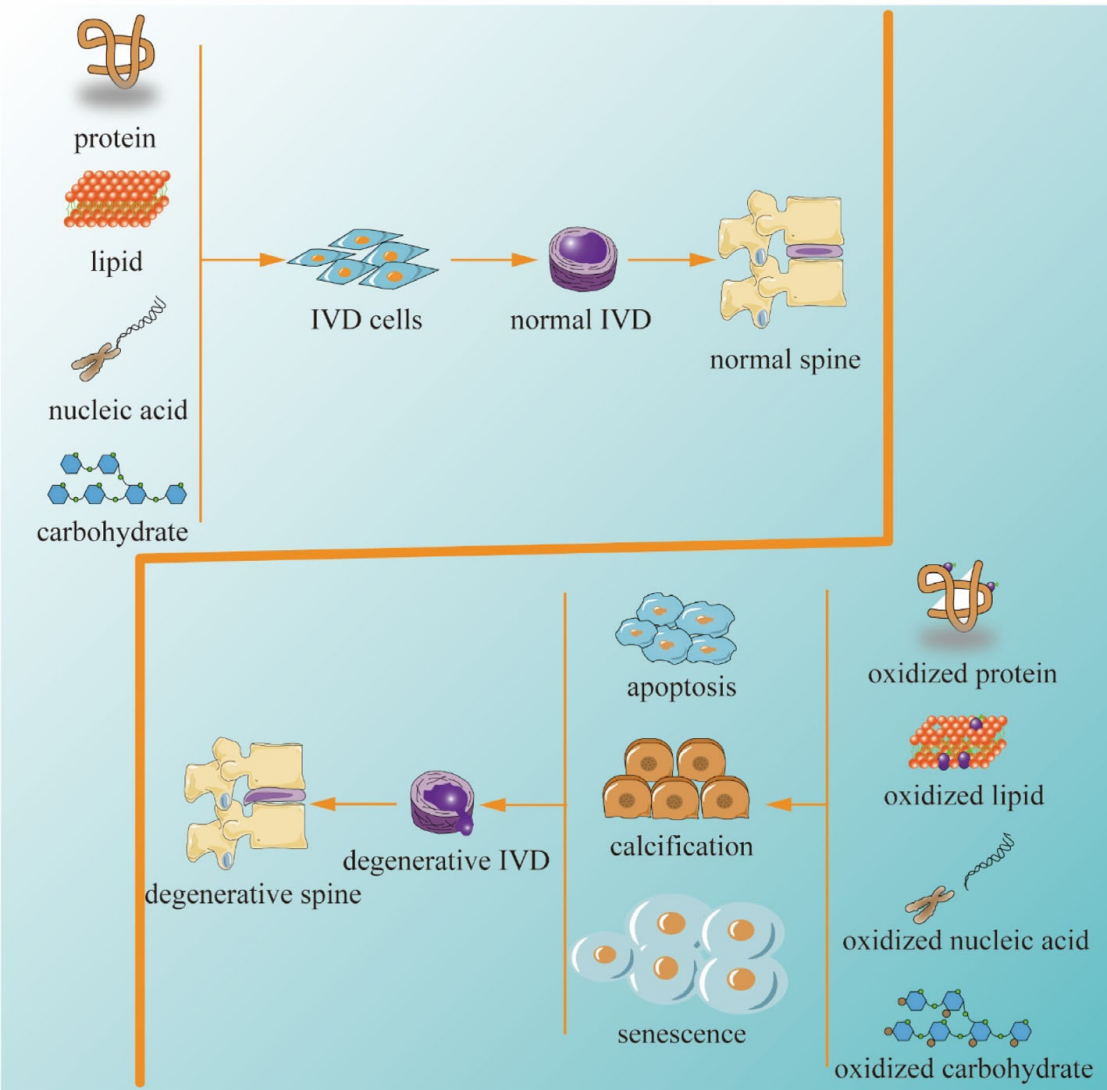
The pathogenic mechanisms of oxidative stress‐induced IDD.

Intervertebral disc degeneration (IDD) is a common age‐related degenerative disease of the musculoskeletal system and is the underlying pathology of disc herniation, spinal stenosis and lumbar spondylolisthesis. Meanwhile, IDD is also a major cause of lower back pain (LBP), which is responsible for approximately 40% of symptomatic LBP^1^ and imposes a serious economic burden on individuals and society by increasing the burden of disease and reducing patient productivity.^2^, ^3^ A conservative estimate is that LBP related direct costs are up to $90 billion annually in the United States alone.^4^ However, the pathogenesis of IDD has not been fully elucidated. Currently, IDD is considered to be a degenerative process involving molecules, cells, tissues, and organs, which leads to significant changes in IVD tissue composition and biomechanical properties, ultimately compromising their ability to withstand loads (Figure 1).^1^

As a spinal connecting device, intervertebral disc (IVD) is mainly composed of nucleus pulposus (NP), fibrous annulus (AF) and cartilage endplate (CEP), which have the functions of buffering spinal pressure and increasing the mobility of the spinal column.^5^, ^6^ IDD, as an age‐related multifactorial disease, remains etiologically incompletely understood to date. However, it is generally believed that genetic susceptibility, age, obesity, smoking, trauma, abnormal non‐physiological mechanical load and other factors contribute to its occurrence and progress.^7^, ^8^, ^9^, ^10^, ^11^, ^12^ During IVD degeneration, a variety of phenotypic changes are involved, including a decrease in the number of NP cells (NPCs), extracellular matrix (ECM) degradation, NP and AF tissues disorganization, CEP calcification and microfractures,^13^, ^14^ which result in reduced IVD height, disc herniation, cartilage calcification, spinal stenosis, and radiculopathy.^3^, ^15^, ^16^ The progression process of IDD may have different stages. First, the structural abnormalities of IVD caused by genetic predisposition, on the basis of which several factors, such as environment, physiology and organisms, cause abnormal IVD microenvironment and cell dysfunction, which can imbalance anabolic and catabolic metabolism and finally lead to IDD.^17^ At present, the clinical treatment of IDD is limited to relieving clinical symptoms through analgesics and surgery, neither of which can solve potential pathological problems by stopping the degeneration process of IVD.^18^, ^19^ Therefore, further exploration of the pathogenic factors and related molecular mechanisms of IDD is of great significance for guiding the treatment of IDD.

Redox balance is important for maintaining normal cellular functions, and its imbalance is involved in various pathological processes that affect human health. Oxidative stress is the result of a dynamic imbalance in redox, manifested by an increase in intracellular reactive oxygen species (ROS) levels in combination with a relative decrease in the levels of antioxidant substances, which can compromise the integrity of cellular functions.^20^, ^21^, ^22^ Current studies have shown that oxidative stress can promote the progression of IDD through multiple pathways,^22^, ^23^ whereas inhibiting the overproduction of ROS within IVD as well as promoting their clearance has been shown to be effective in delaying IDD.^24^, ^25^ Therefore, this review summarizes the potential role of oxidative stress in IDD and discusses the pathophysiological processes and pathogenic mechanisms of IDD, which may provide potential therapeutic strategies for IDD.

## 2 OXIDATIVE STRESS AND IDD

ROS is a kind of unstable and highly active molecules, including superoxide anion (O_2_^−^), hydroxyl radical (OH^−^), hydrogen peroxide (H_2_O_2_) and hypochlorite ion (OC1^−^).^26^ It is a by‐product of cellular aerobic metabolism and is an important intracellular signal molecule at a normal level, which participates in the regulation of various physiological processes in cells.^27^ When it is overproduced, it will damage the cell function and lead to the corresponding disease.^20^, ^28^ Although IVD is in hypoxic environment because there is no direct blood supply,^29^ IVD cells still undergo aerobic metabolism and produce ROS.^30^ Due to its avascular nature,^31^ during IVD degeneration, intracellular metabolites cannot undergo efficient transport, resulting in the accumulation of metabolic waste products that can damage cellular function. In a study by Suzuki et al.,^24^ the level of ROS in human and rat IVD gradually elevated with increasing grades of IVD degeneration. In addition, the level of ROS in NPCs within the degenerating human IVD was positively correlated with the grade of degeneration.^32^ These results all suggest that oxidative stress may be an important factor in promoting the progression of IDD.

## 3 DISTURBANCE OF REDOX STATE IN DEGENERATED IVD

### 3.1 Increased ROS generation

**FIGURE 2:**
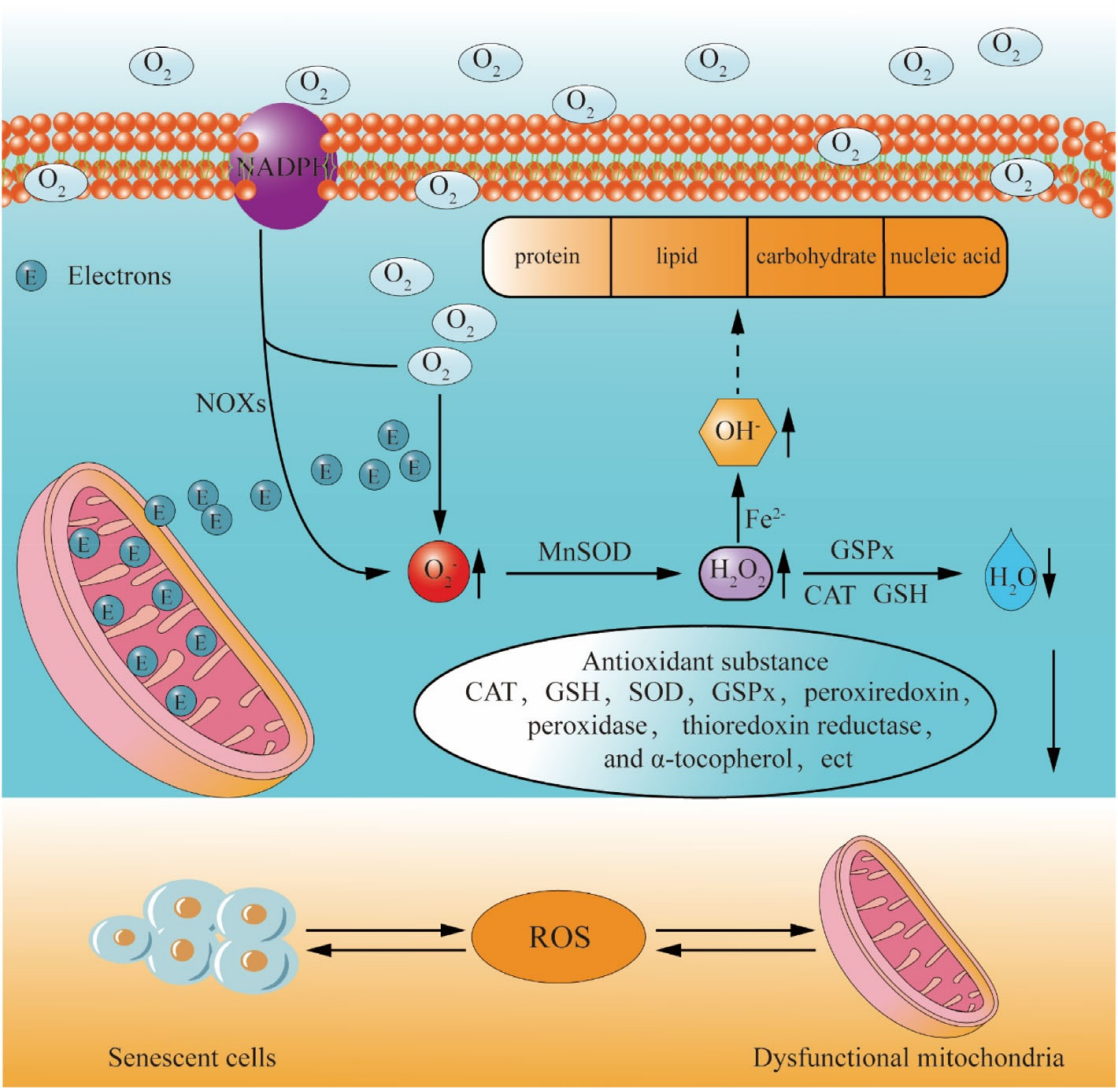
The imbalance of redox homeostasis in IVD cells. In the process of IVD degeneration, the main cell types in NP tissue changed from chondrocyte 1 to chondrocyte 2, chondrocyte 4 and chondrocyte 5. This transformation leads to a change in the main cell types responsible for maintaining redox balance in NP tissue, from chondrocyte 1 to chondrocyte 2, chondrocyte 4 and chondrocyte 5. Oxidative stress is caused by the imbalance between ROS production and clearance in DNP.

There are different cellular compositions in normal NP (NNP) and degenerative NP (DNP) tissues. Li et al.^33^ found that the cells in human NP can be divided into two types: chondrocytes and non‐chondrocytes. The former includes chondrocytes 1, chondrocytes 2, chondrocytes 3, chondrocytes 4 and chondrocytes 5, and the latter includes endothelial cells, macrophages, neutrophils and T cells. After classification was determined by cell cluster analysis, they found that chondrocyte 1 accounted for a higher proportion in NNP tissue, while for DNP tissue, chondrocyte 2, chondrocyte 4 and chondrocyte 5 accounted for a higher proportion. This may imply that the redox microenvironment in NNP tissue and DNP tissue is maintained by different cell types, with chondrocyte 1 being the major cell type responsible for maintaining redox balance in NNP tissue, while chondrocyte 2, chondrocyte 4, and chondrocyte 5 being the major cell types in DNP tissue responsible for maintaining redox balance. As the main intracellular ROS production place, mitochondria are a major site of intracellular ROS production.^34^, ^35^ Under physiological conditions, 0.2%–2% of electrons in the mitochondrial electron transport chain (ETC) do not follow the normal order of transfer, but leak directly from the ETC and interact with oxygen to produce superoxide or H_2_O_2_.^36^ In degenerative IVD, a decline in the recycling capacity of substances and various stress stimuli create a hostile microenvironment,^37^, ^38^ which leads to mitochondrial dysfunction, impairing mitochondrial dynamics and quality control systems, thereby increasing ROS production.^25^, ^39^, ^40^, ^41^ In addition, mitochondria are also the main target of ROS attack. A large amount of ROS can lead to oxidative damage of mitochondrial DNA, lipid and protein, and aggravate mitochondrial dysfunction, thus forming a positive feedback loop.^42^, ^43^ Moreover, cell senescence is also an important reason for the increase of ROS in degenerated IVD.^31^, ^44^ Cellular senescence exhibits a senescence‐associated secretory phenotype (SASP), characterized by the release of various inflammatory cellular factors, growth factors, and enzymes,^45^, ^46^ which is conducive to the production of ROS. For example, ROS production can be increased by the upregulation of NADPH oxidases 4 (NOX4) during cellular senescence.^47^ Similarly, ROS is also the main cause of cell senescence, which forms a vicious circle (Figure 2).^48^, ^49^

### 3.2 Decreased production of antioxidants

The disturbance of redox status in degenerative IVD manifests as an increase in ROS production on the one hand and a decrease in the activity of antioxidant substances on the other. In rat IVD, the level of superoxide dismutase (SOD) decreases with age and degeneration.^50^ In addition, multiple in vitro experiments have shown that the expression levels of SOD, catalase (CAT) and glutathione (GSH) are decreased in degenerated NPCs,^51^, ^52^ which further promote intracellular ROS accumulation. In summary, the imbalance of ROS production and scavenging in degenerated IVD leads to disturbance of redox status (Figure 2).

## 4 OXIDATIVE DAMAGE OF BIOMOLECULES

### 4.1 Oxidative damage of protein

In the organism, proteins are highly susceptible to oxidative damage because they are the most abundant and react rapidly with ROS.^53^ Such damage can lead to changes in the structure, function, turnover and activity of proteins, which can affect the normal function of the cell.^53^, ^54^ Oxidative damage to proteins can be divided into two categories: disruption of the protein backbone and remodelling of amino acid side chains, the former characterized by the fragmentation of the polypeptide chain, and the latter by the formation of a large number of different products.^55^ Among various proteins, the sulphur‐containing amino acids cysteine and methionine are vulnerable to ROS based on the high susceptibility of the electron rich sulphur atoms of their side chains to oxidation.^56^, ^57^ In addition, the aromatic functional groups of amino acids are also excellent targets for oxidative damage. Tyrosine and tryptophan can be oxidized by hydroxyl radicals to 3‐hydroxytyrosine and hydroxytryptophan, respectively.^58^ Another hallmark of protein oxidation is protein carbonylation, which mainly includes three forms: direct oxidation of protein bound amino acids, oxidative cleavage of the protein backbone, and introduction of carbonyl groups from oxidized sugars or oxidized lipids.^59^, ^60^ For example, the basic amino acids lysine and arginine are readily modified by glycosylation.^53^, ^61^, ^62^, ^63^ In addition, oxidative stress can also lead to protein aggregation,^64^, ^65^ which can lead to a variety of pathological conditions in humans.^65^, ^66^ Recently, it has been reported that the levels of advanced oxidative protein products (AOPPs) are significantly higher in human degenerative IVD (Pfirrmann IV or V) tissues than in normal tissues.^67^ Similarly, the level of AOPPs in the IVD of Wistar rats showed age‐related changes.^50^ Further studies revealed that AOPPs can induce phosphorylation of mitogen activated protein kinases (MAPK) in NP and AF cells, which can lead to apoptosis and senescence.^67^, ^68^

### 4.2 Oxidative damage of nucleic acid

In addition to proteins, nucleic acids are also important targets of ROS.^69^ Oxidative damage to DNA and RNA is the result of oxidation of their constituent unit bases, nucleosides, and nucleotides. Among the various bases, guanine (G) is currently the main target for the detection of oxidative nucleic acid damage due to its low oxidation potential, which is most susceptible to ROS.^70^ Among the different G‐oxidation products, 8‐oxoguanine (8‐oxoG) and its corresponding nucleotide 8‐oxo‐2′‐deoxyguanosine (8‐oxodG) are the most predominant forms of oxidative damage to nucleic acids.^71^ 8‐oxoG in DNA results in a tendency to pair adenine(A) instead of cytosine(C), which may cause a C to A substitution.^72^, ^73^ The content of 8‐oxoG in body fluid of patients with oxidative stress‐related psychiatric disorders, chronic kidney disease, gestational diabetes, and neurodegenerative diseases increased.^74^, ^75^, ^76^, ^77^ In addition, a case–control study found that plasma 8‐oxodG levels in patients with lumbar disc herniation were significantly higher than those in healthy controls.^78^ Recently, it was found that the level of 8‐oxodG in human NPCs induced by IL‐1β is significantly increased, while the level of 8‐oxodG is decreased after treatment with antioxidants.^79^ These studies suggest that oxidative damage to nucleic acids may be involved in IDD progression.

### 4.3 Oxidative damage of lipid

In recent years, the potential role of lipid peroxides in various diseases has attracted increasing attention.^80^, ^81^ Lipids are not only an important component of cell membrane, but also play an important role in other aspects of cell structure. Lipid peroxidation can occur through two pathways, enzymatic reaction and nonenzyme dependent reaction.^82^ The former is executed by peroxidases and the latter mainly relies on the iron‐dependent Fenton and Haber‐Weiss reactions and thereby initiates the radical chain reactions required for lipid peroxidation.^83^, ^84^ The main substrates of lipid peroxidation are polyunsaturated lipids, as carbon–carbon double bonds are vulnerable to ROS.^80^ The oxidation process mainly consisted of three stages: initiation, propagation and termination, and the specific process has been described in detail in previous reports.^85^ Most lipid peroxidation products contain carbonyl groups in their structures, and these highly reactive intermediates containing carbonyl moieties are called reactive carbonyl species (RCS).^86^ Compared with free radicals, RCS have a longer lifetime and higher stability, which facilitates their intracellular diffusion and consequent modification of DNA, lipids and proteins.^87^ In a recent study, plasma levels of malondialdehyde (MDA), a lipid peroxidation product, were significantly higher in patients with lumbar disc herniation compared with healthy controls.^78^ In addition, tert‐butyl hydroperoxide (TBHP) and H_2_O_2_ could also increase the MDA level in rat IVD cells.^88^, ^89^, ^90^ These studies all suggest that oxidative stress can lead to lipid peroxidation within IVD, which in turn promotes IDD progression.

### 4.4 Oxidative damage of carbohydrate

In addition to protein, nucleic acid and lipid, carbohydrate is also an important target of ROS.^91^ Under oxidative stress, amino groups in nucleic acids, lipids, and proteins react nonenzymatically with reducing sugars to produce a heterogeneous array of molecules known as advanced glycation end products (AGEs).^92^ AGEs can alter the normal function of nucleic acids, proteins and lipids, resulting in mitochondrial dysfunction.^93^, ^94^ In addition, they can activate a range of receptors, including receptors for AGEs (RAGEs), which trigger downstream pathogenic cascades.^95^, ^96^ A variety of tumours, neurodegenerative diseases, chronic obstructive pulmonary disease, cardiovascular disease, and diabetes have been implicated in the cellular dysfunction caused by AGEs.^97^, ^98^, ^99^ In vitro, AGEs significantly decreased the viability and proliferation of NPCs in a time‐and dose‐dependent manner.^100^, ^101^ In addition, AGEs can lead to excessive production of mitochondrial ROS and prolonged activation of the mitochondrial permeability transitionpore (mPTP) in NPCs, resulting in mitochondrial dysfunction and activation of the mitochondrial apoptotic pathway in human NPCs, which can induce apoptosis by promoting the increase of Bax level and the decrease of Bcl‐2 level.^100^

## 5 OXIDATIVE STRESS AND INTRACELLULAR SIGNAL TRANSDUCTION

**FIGURE 3:**
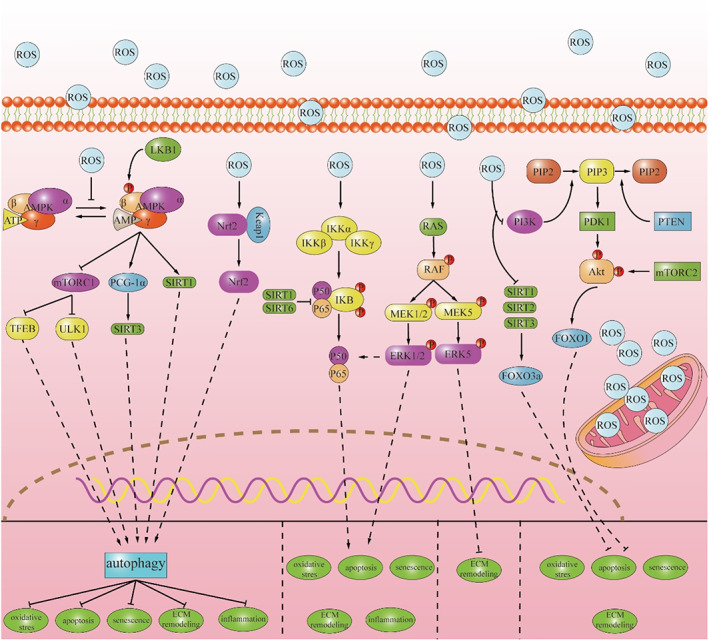
The complex signal networks in degenerative IVD cells.

ROS, as an intracellular signal molecule, participates in complex intracellular signal transduction. Since ROS are involved in various cellular processes such as apoptosis, senescence, autophagy and inflammatory response, it can be speculated that ROS modulate the phenotypic changes of IVD cells through complex signalling networks composed of different signalling pathways, either directly or indirectly (Figure 3).^26^ However, our understanding of the role of ROS in the signalling network of IVD cells is limited. Further insight is needed to elucidate more signalling pathways regulated by ROS and their direct mechanisms.

### 5.1 Keap1‐Nrf2‐ARE

Nrf2 is a transcription factor that enhances cellular defence systems against oxidative stress and inflammatory responses. In the resting state, Nrf2 binds to Kelch‐like ECH‐associated protein 1 (Keap1) in the cytoplasm and is strictly negatively regulated by Keap1.^102^ Keap1 mediates Nrf2 ubiquitin‐dependent proteasomal degradation in the cytoplasm by acting as an articulation molecule for the CUL‐E3 ligase.^102^, ^103^ Under oxidative stress, the dissociation of Keap1 and CUL‐E3 ligase leads to the conformational change of Keap1 and the release of Nrf2, which leads to the accumulation of Nrf2 in the cytoplasm and subsequent nuclear transfer.^104^ After entering the nucleus, Nrf2 binds to the antioxidant response element (ARE) and promotes the transcription of antioxidant genes, including heme oxygenase‐1 (HO‐1), NAD (P) H dehydrogenase quinone 1 (NQO1), and ferritin, to maintain the intracellular redox balance.^105^ Currently, an increasing number of studies have shown that activation of the Keap1‐Nrf2‐ARE signalling pathway is effective in delaying IDD. In human NP tissues, the expression level of Nrf2 was negatively correlated with Pfirrmann grade.^89^ Similarly, Nrf2 expression levels were significantly decreased in the degenerative NP tissues of rats induced by acupuncture and compression.^25^, ^89^ These results suggest that decreased Nrf2 levels correlate with the severity of IDD. Recently, Kang et al.^106^ found that the long non‐coding RNAs (lncRNA) ANPODRTk could alleviate TBHP‐induced oxidative stress and apoptosis in NPCs by activating Nrf2 signalling, while Nrf2 knockdown attenuated the protective effect of ANPODRT. Mechanistically, ANPODRT acted by promoting the nuclear translocation of Nrf2 through breaking the keap1‐nrf2 link. In addition, activation of Nrf2 signalling increases antioxidant enzymes, including nitric oxide synthase (iNOS), NOX4, SOD2 and LDH, and ECM synthesis under H_2_O_2_ induction.^107^, ^108^ Nrf2 signalling also regulates autophagy. Tang et al.^109^ showed that Nrf2 knockdown reduced autophagy‐related protein (ATG) expression, which exacerbated H_2_O_2_‐induced IVD degeneration. Furthermore, icariin (ICA) inhibited H_2_O_2_‐induced mitochondrial dysfunction, oxidative stress and apoptosis in CEP cells by activating Nrf2/HO‐1 signalling axis, while Nrf2 knockdown reversed the protective effects of ICA.^110^, ^111^ In conclusion, activation of the keap1‐Nrf2‐ARE signalling pathway can inhibit oxidative stress and apoptosis and activate autophagy in IVD cells to delay IDD.

### 5.2 PI3K‐Akt

Phosphatidylinositol‐3‐kinase (PI3K)/protein kinase B (Akt) signalling pathway is an important intracellular signalling pathway with critical regulatory roles in apoptosis, growth and metabolism.^112^ Various molecules including cytokines, glucose, insulin, and drugs can initiate PI3K/Akt signalling.^113^ These molecules lead to the conversion of phosphatidylinositol4,5‐bisphosphate (PIP2) to PIP3 by PI3K, which further activates Akt and its downstream effector molecules to regulate cellular function.^114^ In this process, phosphatase and tensin homologue (PTEN) negatively regulates the PI3K/Akt signalling pathway by converting PIP3 to PIP2.^115^ ROS can inhibit its downstream signal transduction by inhibiting the phosphorylation of PI3K and Akt and promoting PTEN expression.^116^, ^117^ In IDD, activation of the PI3K/Akt signalling pathway effectively ameliorated oxidative stress‐induced apoptosis, ECM degradation, and cell proliferation of NPCs^118^, ^119^ .In addition, Guo et al.^120^ found that resveratrol (RSV) could promote ECM production and increase the expression of autophagy related markers (beclin‐1 and LC‐3) to induce cell autophagy to delay IDD. Mechanistic studies revealed that RSV exerted a protective effect through activation of the PI3K/Akt pathway. Recently, it has been reported that 1α,25‐dihydroxyvitamin D_3_(1,25(OH)_2_D_3_)and naringin (Nar) inhibit H_2_O_2_‐induced apoptosis and mitochondrial dysfunction of nucleus pulposus‐derived mesenchymal stem cells (NPMSCs) by activating PI3K/Akt signalling pathway.^121^, ^122^ These lines of evidence suggest that there are complex regulatory mechanisms between ROS and PI3K/Akt signalling pathway, and activating PI3K/Akt signalling pathway may effectively inhibit oxidative stress to delay IDD progression.

### 5.3 AMPK

As a classical intracellular signalling pathway, the AMP‐activated protein kinase (AMPK) signalling pathway plays an important role in regulating cell proliferation, apoptosis, autophagy and differentiation under pathophysiological conditions.^123^ In the resting state, AMPK is bound to ATP in an inactive state.^124^ Under energy deprivation or stress, the ratio of intracellular AMP: ATP or ADP: ATP increases, which leads to the binding of AMP to the γ subunit of AMPK and triggers the conformational change leading to the first phosphorylation of AMPK.^125^ Subsequently, active kinase B1 phosphorylates threonine(Thr) 172 in the α subunit to further activate AMPK.^126^ In addition, intracellular transforming growth factor‐β‐activated kinase1 and calcium‐dependent protein kinase β can also phosphorylate Thr172 in the α subunit leading to activation of AMPK.^127^, ^128^ At present, many studies have shown that AMPK signal pathway can regulate a variety of important biological behaviours of IDD. Lin et al.^129^ and Song et al.^100^ found that activation of the AMPK/peroxisome proliferator‐activated receptor‐γ coactivator 1α (PGC‐1α) signalling axis ameliorated oxidative stress‐induced apoptosis, senescence and mitochondrial redox homeostasis disorders in NPCs through upregulation of SIRT3. In addition, curcumin (CUR) inhibited TBHP‐induced oxidative stress and mitochondrial dysfunction, which contributed to the attenuation of apoptosis, senescence and ECM degradation in human NPCs. Mechanistic studies revealed that CUR induced autophagy to attenuate oxidative damage and delay IDD in NPCs in an AMPK/mTOR/ULK1 dependent manner.^130^ Recently, Zhang et al.^131^ found that orientin (Ori) ameliorates TBHP‐induced oxidative stress, apoptosis, mitochondrial dysfunction and endoplasmic reticulum (ER) stress in NPCs by activating the SIRT1/AMPK signalling axis. These pieces of evidence suggest that AMPK signalling pathway plays an important role in IDD, and the activation of this pathway is expected to delay IDD.

### 5.4 NF‐κB

NF‐κB belongs to a family of transcription factors with two distinct activation mechanisms, canonical and noncanonical, the former of which is involved in the regulation of inflammation, immune response, cell proliferation and survival.^132^ In the resting state, NF‐κB binds to its specific inhibitor IκB and exists in the cytoplasm as an inactive complex.^133^ IκB mainly consists of IKKα (IKK1), IKKβ (IKK2) and the regulatory subunit IKKγ, which forms the IKK complex with IKKα/IKKβ as a dimer. When cells are stimulated by inflammatory factors, oxidative stress and mechanical stress, IκB is phosphorylated and degraded by ubiquitin‐dependent proteasome.^134^ Subsequently, NF‐κB releases κB transcription factors into the nucleus to activate downstream target genes to regulate cellular functions.^135^ Studies have shown that ROS can lead to the phosphorylation of IKKα, which leads to the classical activation pathway of NF‐ κB.^136^ Similarly, ROS can also affect the activity of IKKβ by mediating its S‐glutathionylation, which in turn promotes the nuclear translocation of NF‐κB.^137^ In addition, AGEs can promote the activation of NF‐κB signalling by binding to RAGEs.^138^ In vitro, H_2_O_2_ treatment can induce the activation of NF‐ κ B pathway, resulting in NPCs apoptosis and ECM degradation.^139^ Consistent with this, multiple studies have shown that increased intracellular ROS can activate NF‐κB pathway and lead to NPCs inflammation, senescence, apoptosis, ECM degradation and mitochondrial dysfunction, while inhibition of NF‐κB pathway can reverse this phenomenon and delay IDD.^39^, ^79^, ^140^, ^141^ In addition, ROS can also activate NF‐κB pathway in AF and CEP cells, leading to cell damage and promoting IDD.^142^, ^143^ These studies suggest that the abnormal activation of NF‐κB signalling pathway seriously affects the survival and function of IVD cells in oxidative stress microenvironment, and blocking this pathway is expected to retard oxidative stress‐induced IDD.

### 5.5 MAPK/ERK

In mammals, the mitogen‐activated protein kinase (MAPK)/extracellular signal‐regulated kinase (ERK) signalling pathway, also known as the RAS–RAF–ERK–MAPK signalling pathway, consists of three RAF proteins (RAF1, A‐and B‐RAF), two MEK proteins (MEK1 and ‐2) and two ERK proteins (ERK1 and ‐2).^144^ There are three major MAPK cascades in eukaryotic cells: ERK, c‐Jun NH2‐terminal kinase (JNK) and p38, which regulate cell survival, death, differentiation, proliferation and metabolism through phosphorylation of downstream substrates.^126^ Activation of MAPK/ERK signalling pathway is closely related to tumour, neurodegenerative and infectious diseases, and also plays an important role in the development and progression of IDD.^145^, ^146^, ^147^ Seol et al.^148^ showed that TBHP significantly increased mitochondrial ROS production in NPCs and activated ERK, JNK and p38 signalling pathways to promote apoptosis. In addition, MAPK/ERK signalling also accelerated IDD by inhibiting autophagy and promoting ECM degradation in NPCs.^149^ It has been reported that H_2_O_2_ can increase CEP apoptosis and CEP calcification by stimulating the ROS/MAPK/NF‐κB signalling axis.^143^ Furthermore, the phosphorylation level of p38MAPK in degenerated IVD of rats induced by acupuncture significantly increased.^150^ Interestingly, ERK5 may play opposing roles in IDD compared with ERK1/2. Liang et al.^151^ showed that the level of ERK5 in degenerative NP tissues was lower than that in normal tissues. SiRNA‐mediated ERK5 knockdown and inhibition of the ERK5 inhibitor BIX02188 resulted in reduced expression levels of extracellular matrix of NPCs, which suggested that inhibition of ERK5 might accelerate IDD progression. These results suggest that selective activation of the MAPK/ERK signalling pathway under specific conditions of stress induction context, timing and extent leads to differences in the expression of oxidative stress, catabolic and apoptotic phenotypes within the IVD.

### 5.6 SIRT

The Sirtuin (SIRT) protein family includes seven members from SIRT1 to SIRT7, all of which have highly conserved nicotinamide adenine dinucleotide+ (NAD+) binding and catalytic domains with distinct cellular localizations.^152^ SIRT1, SIRT6 and SIRT7 are mainly located in the nucleus, SIRT3, SIRT4 and SIRT5 are distributed in the mitochondria, and SIRT2 is mainly in the cytoplasm.^153^ SIRT family proteins are functionally similar, with SIRT1‐SIRT3 having strong deacetylase activity and SIRT4‐SIRT7 being weaker.^152^ SIRT plays an important role in many diseases by regulating downstream target genes to mediate cell survival, anti‐inflammatory response and anti‐oxidative stress.^154^, ^155^ At present, SIRT1‐SIRT3 have been most extensively studied in IDD. Ma et al.^156^ found that SIRT1 overexpression ameliorated IL‐1β Induced mitochondrial dysfunction and ROS accumulation in NPCs, and inhibited NLRP3 inflammasome activation and apoptosis by promoting PINK1/parkin mediated mitophagy. In addition, activation of SIRT1 can ameliorate oxidative stress mediated p53‐p21‐Rb and p16‐Rb related cellular senescence.^157^, ^158^, ^159^ In addition to SIRT1, SIRT2 also inhibits oxidative stress‐induced IVD cell injury. Yang et al.^160^ found that SIRT2 overexpression inhibited oxidative stress by upregulating the expression of SOD1/2 and suppressed NPCs senescence by downregulating p53‐p21‐Rb levels. Moreover, in rat AF cells, TBHP inhibited autophagy and promoted apoptosis in a time‐and dose‐dependent manner, which was further aggravated by SIRT2 knockdown.^161^ Similar to SIRT1 and SIRT2, activating SIRT3 also delays IDD progression. Multiple studies have found that activation of AMPK/PGC‐1α Pathway can promote SIRT3 expression, which can ameliorate oxidative stress‐induced senescence, apoptosis and ECM degradation in NPCs.^100^, ^129^ In addition, Zhou et al.^162^ reported that SIRT3 overexpression promoted the synthesis of SOD2 to restore the redox balance within IVD by activating the transcription factor FOXO3a. Similarly, SIRT6 overexpression could also attenuate oxidative stress‐induced senescence and apoptosis of NPCs by upregulating autophagy.^163^ These studies demonstrate that the SIRT family has an important role in maintaining redox balance and holds promise as a new target for IDD therapy.

### 5.7 mTOR

Mammalian target of rapamycin (mTOR), as a protein kinase that responds to nutrient levels and growth signals, is a central signalling molecule that integrates growth and metabolism.^164^ mTOR plays an important role in various degenerative diseases such as osteoarthritis, diabetes, atherosclerosis and Parkinson's disease through its involvement in the regulation of protein synthesis, cellular senescence, autophagy, apoptosis and immunity.^165^, ^166^ Recently, studies have shown that mTOR signalling is essential for maintaining IVD homeostasis.^167^ Kang et al.^130^ showed that CUR increased LC3‐II/LC3‐I ratio and Beclin‐1 levels and decreased P62 levels by regulating AMPK/mTOR/ULK1 signalling pathway in NPCs, which in turn inhibited TBHP‐induced apoptosis, senescence and ECM degradation in NPCs by promoting autophagy. In addition, EB transcription factor EB (TFEB) is the main transcription regulator of lysosome and autophagy genes.^168^ Apigenin can promote the nuclear translocation of TFEB by down‐regulation of mTOR signalling pathway and promote autophagy to protect NPCs from TBHP induced oxidative damage.^169^ Recently, it was found that inhibition of the mTOR/p70S6K signalling pathway promoted autophagy in AF and CEP cells and attenuated oxidative stress caused by H_2_O_2_.^170^, ^171^ These results suggest that inhibition of mTOR signalling pathway can effectively promote autophagy, which may contribute to the survival of IVD cells and the maintenance of homeostasis of the microenvironment in IVD.

## 6 MECHANISM OF OXIDATIVE STRESS IN IDD

### 6.1 Oxidative stress and cell death

The maintenance of normal IVD function depends on a sufficient number of cells and the metabolic capacity endowed by normal cell function. The excessive death of IVD cells leads to a decrease in the number of cells with normal functions, which leads to the imbalance between anabolism and catabolism, and finally leads to IDD. At present, studies have found that oxidative stress is one of the important factors that promote IVD cell death and thus accelerate IDD.^25^, ^172^

#### 6.1.1 Oxidative stress and apoptosis

Apoptosis is the process by which cells stop growing and dividing, ultimately leading to controlled cell death.^173^ Apoptosis is divided into intrinsic and extrinsic apoptosis, with the former also known as mitochondrial pathway apoptosis. The activation of intrinsic apoptosis relies on the aberrant activity of Bcl‐2 family proteins on the mitochondrial membrane, which leads to increased permeability of the outer mitochondrial membrane, leakage of cyt‐c into the cytosol,^174^ and subsequent activation of a series of caspases that ultimately induce apoptosis.^173^, ^174^ Among the numerous factors that induce intrinsic apoptosis, ROS is an important member.^89^, ^175^ Both H_2_O_2_ and TBHP significantly increase ROS production and the leakage of cyt‐c from the mitochondrial intermembrane space to the cytosol, and ultimately promote apoptosis by promoting Bax expression and inhibiting Bcl‐2 expression.^90^, ^176^ In addition, compression treatment can significantly increase the production of ROS in the cytoplasm and mitochondria of NPCs, which is accompanied by mitochondrial dysfunction and the decrease of Nrf2 signal level, and ultimately leads to apoptosis through the mitochondrial pathway.^25^ Recent studies have found that tert‐butylhydroquinone can inhibit TBHP induced apoptosis of rat NPCs by upregulating the activity of Nrf2/SIRT3 pathway, and delay the progress of IDD.^89^ These studies suggest that oxidative stress can promote IDD, while inhibition of oxidative stress can delay IDD by reducing apoptosis of IVD cells.^39^, ^177^

#### 6.1.2 Oxidative stress and pyroptosis

Unlike conventional apoptosis, pyroptosis is closely associated with the inflammatory response and is also known as pro‐inflammatory programmed cell death.^178^, ^179^, ^180^ Oxidative stress‐induced pyroptosis is partly dependent on the bridging role of NLRP3 inflammasome based on the fact that ROS can promote the assembly and activation of NLRP3 inflammasome.^181^ Activated NLRP3 inflammasome can cleave pro‐caspase‐1 into caspase‐1, which can induce pyroptosis.^182^, ^183^ Therefore, NLRP3 inflammasome and caspase‐1 can also be considered as markers of pyroptosis. Compared with normal cells, the contents of ROS and caspase‐1 in primary NPCs of degenerative human IVD tissue were significantly increased.^32^ Similarly, pretreatment of NPCs with H_2_O_2_ can increase the expression of ROS, NLRP3 inflammasome and caspase‐1 in cells, indicating that oxidative stress can effectively induce pyroptosis.^32^ Moreover, it was found that co‐culture of P. acnes and H_2_O_2_ with NPCs induced the overexpression of ROS, caspase‐1 and NLRP3, and promoted pyroptosis in NPCs via thioredoxin interaction protein (TXNIP) /NLRP3 pathway, which was significantly attenuated after inhibition of oxidative stress.^184^, ^185^ Combined with the above evidence, ROS could promote pyroptosis by activating NLRP3 inflammasome, while inhibition of oxidative stress could effectively inhibit pyroptosis to retard IDD.

#### 6.1.3 Oxidative stress and ferroptosis

As a ubiquitous non‐apoptotic form of cell death, ferroptosis has attracted the attention of a wide range of researchers in recent years.^186^, ^187^ It has been found that the redox imbalance is the main cause of ferroptosis, which is related to the overexpression and abnormal activation of many oxidoreductases involved in the production and clearance of ROS.^188^ Therefore, ferroptosis is induced by ROS and precisely regulated at multiple levels including transcription, translation and post‐translational modifications.^189^, ^190^, ^191^ In a study by Yang et al.,^88^ glutathione peroxidase 4 (GPx4) and ferritin heavy chain (FTH) expression were decreased and prostaglandin endoperoxide synthase 2 (PTGS2) expression was elevated in human degenerative IVD tissues compared with normal controls, suggesting that ferroptosis is increased in degenerative IVD. In vitro, after treatment of rat NPCs with TBHP, the expression of FTH and GPx4 decreased and PTGS2 expression increased in a dose‐dependent manner. In addition, Zhang et al.^192^ showed that homocysteine (Hcy) can upregulate oxidative stress and ferroptosis levels in rat NPCs by promoting GPX4 methylation. Recently, iron overload has been found to be an independent risk factor for human IDD, which promotes CEP calcification and CEP cell ferroptosis, leading to IDD progression.^193^ Furthermore, Lu et al.^194^ found that intercellular iron overload mediated by dysfunction of ferroportin (FPN) played an important role in TBHP‐induced ferroptosis in human NPCs, while increased nuclear translocation of metal‐regulatory transcription factor 1 (MTF1) restored the function of FPN, eliminated intercellular iron overload, and protected cells from ferroptosis. These results suggest that oxidative stress can induce iron death and that mitigating oxidative stress contributes to the inhibition of ferroptosis in IVD cells.

### 6.2 Oxidative stress and cell senescence

Cell senescence is a stable cell cycle arrest that occurs in diploid cells and limits their ability to proliferate,^195^ which can be divided into replicative senescence and stress‐induced senescence.^196^, ^197^ Stress‐induced senescence is mediated by a range of internal or external, physical or chemical, acute or chronic factors, independent of telomere length.^198^ Among these factors, ROS play an important role and can lead to disruption of cell membrane structure, permeability changes and cytotoxic responses when ROS levels exceed the antioxidant capacity of the cell.^44^, ^199^ Over time, oxidative damage accumulates and contributes to aging and various degenerative diseases.^200^, ^201^

Previous studies have shown that ROS levels in human and rat IVD increase progressively with the degree of IVD degeneration.^24^ Histological analysis of human IVD specimens showed that the proportion of Senescence‐Associated β‐Galactosidase (SA‐β‐gal) positive cells in Pfirrmann IV/V IVD was significantly higher than that in Pfirrmann I/II, and highly expressed p53, p21 and pRb.^202^ Similarly, the ratio of SA‐β‐gal positive cells increased in aged gerbil IVD.^203^ In addition, the proportion of senescent cells in IVD of patients with lumbar disc herniation is significantly higher than that in spondylolisthesis and scoliosis IVD, which may be related to the fact that IVD cells obtain more oxygen for aerobic respiration through vascularization within adjacent tissues or the herniation itself.^204^ These in vivo studies suggest that excessive cellular senescence in IVD may be associated with oxidative stress. In vitro, most of the cells treated with H_2_O_2_ for NPCs 10 days showed positive SA‐β‐gal and highly expressed p53 protein.^205^ Moreover, high glucose stress could increase ROS generation in AF cells in a dose‐and time‐dependent manner and induce cell senescence by activating the p16/Rb signalling axis, whereas inhibition of oxidative stress significantly alleviated cell senescence.^206^ These results illustrated that oxidative stress could promote IVD cell senescence, while multiple studies confirmed that antioxidant treatment could effectively inhibit oxidative stress‐induced cell senescence to delay IDD progression.^169^, ^207^, ^208^

### 6.3 Oxidative stress and autophagy

Autophagy is an evolutionarily conservative self‐degradation system that captures and degrades misfolded proteins and damaged organelles to circulate intracellular components to maintain intracellular homeostasis under stress.^209^, ^210^ In recent years, increasing evidence has demonstrated that ROS are important intracellular signal transducers for the maintenance of autophagy.^211^ Autophagy in normal cells is in dynamic equilibrium, and disruption of this equilibrium can affect cell function and survival.

At present, some studies suggest that autophagy disorder in IVD cells may be an important factor in IDD.^167^, ^212^ TBHP treatment resulted in decreased levels of LC3II/I and increased levels of p62 in NPCs, indicating that autophagic flux decreased under oxidative stress, while intervention with autophagic agonists restored autophagic flux and inhibited degeneration of NPCs.^130^, ^213^ In addition, Chen et al.^214^ also showed that the autophagy agonist Nar increased autophagic flux and protected human NPCs from TNF‐α‐induced inflammatory, oxidative stress damage by activating the AMPK/SIRT1 signalling axis. However, it is interesting to note that autophagy not only protects IVD cells from oxidative stress‐induced damage, but also plays a role in promoting the IDD process. In the rat model, autophagy activity was significantly elevated in degenerative NP and AF cells compared with normal cells.^215^, ^216^ Tang et al.^109^ found that H_2_O_2_ treatment can lead to increased expression of autophagosomes and autophagy related markers within rat NPCs, which may accelerate cell death. In addition, the expression of ROS and autophagy related markers beclin‐1, LC3‐II, ATG3, 5, 7 and 12 in notochord cells treated with high glucose increased in a dose‐and time‐dependent manner.^217^ In summary, under oxidative stress, appropriate autophagy activity is conducive to the survival of IVD cells, while excessive autophagy will lead to the degradation of normal organelles and proteins, thus increasing the possibility of cell death and accelerating the process of IDD.

### 6.4 Oxidative stress and ECM remodelling

The mechanical function of the IVD relies on the integrity of its tissue structure. In degenerative IVD, the catabolism of ECM increases and the anabolism decreases due to the decrease of cell number and abnormal function.^218^, ^219^ In addition, aggrecan (Agg) and collagen II (Col II) were gradually replaced by Col I, which changed the structure and biomechanical properties of IVD.^220^ It has been found that oxidative stress is involved in the transformation of metabolic state and components of ECM. After treatment of rat and human NPCs with H_2_O_2_, the expression of ECM component proteins Col II and Agg decreased significantly, while the expression of pro‐degradation proteins, including matrix metalloproteinase‐3 (MMP‐3), MMP‐13, a disintegrin and metalloproteinase with thrombospondin motifs‐4 (ADAMTS‐4) and ADAMTS‐5, increased significantly.^90^, ^129^, ^221^, ^222^ Similarly, oxidative stress also promotes catabolism and inhibits anabolism in AF cells.^24^, ^223^ Moreover, oxidative stress can also cause oxidative damage to ECM related molecules, further reducing ECM content.^224^ In summary, oxidative stress can lead to abnormal extracellular matrix metabolism in IVD and oxidative damage in ECM, promoting IDD progression.

### 6.5 Oxidative stress and inflammation

At present, studies have found that inflammatory mediators and related signalling pathways are important factors in promoting the occurrence and development of IDD.^14^, ^225^ IDD is often accompanied by infiltration of inflammatory cells (mast cells, macrophages, and neutrophils) that secrete a variety of cytokines, including TNF, IL‐1 α, IL‐1 β, IL‐6, IL‐17, IL‐8, IL‐2, IL‐4, IL‐10, interferons‐γ(IFN‐γ) and prostaglandin E2 (PGE2), and participate in multiple key pathophysiological processes.^226^, ^227^ TNF‐ α and IL‐1β can significantly increase the production of monocyte chemoattractant protein‐1, IL‐6, IL‐8 and PGE2 in IVD cells, leading to an inflammatory cascade and a persistent local inflammatory microenvironment by means of positive feedback between proinflammatory cytokines.^14^, ^167^ In addition, pro‐inflammatory cytokines can promote the expression of ECM degrading enzymes (MMPs, ADAMTS), cell senescence and death in the IVD, leading to ECM degradation and destruction of the IVD structure.^228^, ^229^ Furthermore, the inflammatory reaction can induce the infiltration of blood vessels and nerve endings, which further aggravate the deterioration of IVD microenvironment and IVD derived LBP.^226^, ^230^

Oxidative stress is one of the main trigger factors of inflammatory reaction in degenerative IVD.^230^ H_2_O_2_ intervention significantly promoted the expression of IL‐1β, IL‐6, TNF‐α and nitric oxide synthase (iNOS) in rat NPCs.^221^ In addition, ROS can aggravate inflammation by inducing mitochondrial dysfunction and promoting NLRP3 activation.^185^, ^231^, ^232^, ^233^ Oxidative stress can promote inflammation, and inflammation can in turn exacerbate oxidative stress. TNF‐ α and IL‐1β can induce oxidative stress by promoting the expression of ROS and inhibiting the expression of SOD in NPCs.^39^, ^116^, ^214^ These pieces of evidence suggest that there is a complex regulatory network between oxidative stress and inflammation, which accelerates the progress of IDD.

## 7 THERAPEUTIC TARGETS OF OXIDATIVE STRESS‐RELATED IDD

### 7.1 Natural molecule

Many natural substances have antioxidant activity and have been studied in IDD. Nar is a bioflavonoid derived from tomatoes, grapefruit and citrus that has been found to have a variety of biological effects, including antioxidant, anti‐inflammatory and anti‐apoptotic.^122^ Nar treatment can maintain redox homeostasis in NPCs by restoring mitochondrial transmembrane potential (*ΔΨ*m) levels, increasing ATP production, promoting antioxidant expression, and inhibiting ROS production.^214^ Nar also regulates the expression of Col II, Agg, MMP‐3, MMP‐13 and ADAMTS‐4 to maintain ECM quality.^214^ In addition, apoptosis and inflammatory response are important pathogenic factors of oxidative stress‐induced IDD. Nar suppresses inflammatory responses through downregulation of COX‐2 expression and inhibits oxidative stress‐induced mitochondrial pathway apoptosis through upregulation of Bcl‐2 as well as downregulation of cleaved‐caspase‐3 and Bax.^214^, ^234^ Related mechanistic studies revealed that NAR could enhance autophagic flux by activating the AMPK signalling pathway, thereby protecting NPCs from oxidative stress injury.^214^, ^234^ In addition to Nar, quercetin (Que) is also a member of the natural flavonoid family with anti‐inflammatory, anti‐aging and antioxidant properties.^235^ It has been found that Que can promote autophagy and alleviate TBHP‐induced NPCs apoptosis and ECM degradation by regulating SIRT1 and p38 MAPK/mTOR signal pathways. Importantly, quercetin has also been shown to reduce acupuncture‐induced IDD progression in rats in vivo.^149^, ^213^ In addition, salvianolic acid B (SAB), as the most abundant water‐soluble compound in Danshen, has excellent antioxidant properties.^236^ SAB can ameliorate H_2_O_2_‐induced oxidative stress injury in NPCs by promoting the activation of the Janus kinase 2 (JAK2)/signal transducer and activator of transcription 3 (STAT 3) signalling pathway. In vivo, SAB significantly improved the imaging and histological changes of acupuncture‐induced IDD in rats after 6 weeks of treatment.^177^ Moreover, mangiferin (MGF), a natural C‐glucoside xanthone isolated from plants, plays a role in antagonizing oxidative stress and ameliorating mitochondrial dysfunction in a variety of diseases.^237^ Recently, Yu et al.^39^ found that MGF effectively inhibited nuclear translocation of NF‐κB, which in turn attenuated oxidative stress‐induced mitochondrial dysfunction, inflammatory response, ECM degradation and apoptosis in NPCs, and delayed the progression of IDD(Table 1).

**TABLE 1:** Therapeutic targets of oxidative stress‐related IDD

Classification	Antioxidant	Experimental models (stimuli)	Function	Signal pathway	References
Natural molecular	Nar	Human, rat NPCs(TNF‐α, TBHP)	ROS↓ apoptosis↓ catabolism↓ inflammatory↓ autophagy↑	AMPK↑	^214^, ^234^
	Que	Rat NPCs (TBHP)	ROS↓ apoptosis↓ catabolism↓ autophagy↑	SIRT1↑MAPK/mTOR↓	^149^, ^213^
	SAB	Rat NPCs (H_2_O_2_)	ROS↓ apoptosis↓ proliferation↑	JAK2/STAT3↑	^177^
	MGF	Human NPCs (TNF‐α)	ROS↓ Δ*Ψ*m↑ apoptosis↓ catabolism↓ inflammatory↓	NF‐κB↓	^39^
ROS scavenger	MitoQ	Human NPCs (compression)	ROS↓ ΔΨm↑ mPTP↓ apoptosis↓ mitophagy↑	PINK1/Parkin↑Nrf2↑	^25^
	GSH	Human NPCs (H_2_O_2_)	ROS↓ apoptosis↓ catabolism↓	–	^241^
	NAC	Human CEP cell (H_2_O_2_)	ROS↓ apoptosis↓	MAPK↓NF‐κB↓	^143^
Hormone	MT	Rat NPCs (H_2_O_2_, TBHP)	ROS↓ apoptosis↓ catabolism↓ autophagy↑	PINK1/Parkin↑	^90^, ^176^
	E2	Rat (ovariectomized)	Antioxidant enzymes↑ autophagy↓	—	^52^
	PTH	Human NPCs (IL‐1β)	ROS↓ apoptosis↓ catabolism↓ inflammatory↓	CREB/SHH↑	^247^
	CST	Human NPCs(IL‐1β, TNF‐α)	ROS↓ apoptosis↓ catabolism↓ inflammatory↓	AMPK/PGC‐1α↑ NF‐κB/NLRP3↓	^22^
Medicine	1,25(OH)_2_D_3_	Rat AF cell (H_2_O_2_), mouse NPCs (IL‐1β), rat NPMSCs(H2O2)	ROS↓ apoptosis↓ catabolism↓ autophagy↓ senescence↓	mTOR/p70S6K↑ PI3K/Akt↑ NF‐κB↓	^121^, ^141^, ^171^
	MF	Rat NPCs(TBHP)	ROS↓ apoptosis↓ catabolism↓ autophagy↑ senescence↓	AMPK↑ cGAS‐STING↓	^208^, ^249^
	PGZ	Human NPCs (compression)	ROS↓ apoptosis↓ proliferation↑	–	^250^
	Aspirin	Rat NPCs (LPS)	ROS↓ catabolism↓ inflammatory↓	AMPK↑	^251^
	Ulinastatin	Human NPCs (IL‐1β)	ROS↓ apoptosis↓ catabolism↓ inflammatory↓	Nrf2↑NF‐κB↓	^252^
	Amobarbital	Rabbit NPCs (TBHP)	ROS↓ apoptosis↓ necrosis↓	Nrf2↑MAPK↓	^148^
Biomaterials	PBNPs	Rat NPCs (H_2_O_2_)	ROS↓ catabolism↓ inflammatory↓ proliferation↑	–	^255^
	PBNPs@OBG	Rat NPCs (H_2_O_2_)	ROS↓ apoptosis↓ catabolism↓	–	^256^
	Rapa@Gel	Rat (acupuncture)	ROS↓ catabolism↓ inflammatory↓	–	^257^
Stem cell therapy	MSC‐exos	Rat NPCs (H_2_O_2_)	ROS↓ apoptosis↓ catabolism↓ inflammatory↓	–	^259^
	MSC‐exos	Rat CEP cell (TBHP)	ROS↓ apoptosis↓ calcification↓ ER‐Stress↓	ATF6↓	^260^
	CESC‐exos	Rat NPCs (TBHP)	ROS↓ apoptosis↓ autophagy↑	PI3K/Akt↑	^261^
Others	HIF‐1α	Mouse NPCs (TNF‐α)	ROS↓ apoptosis↓ catabolism↓ inflammatory↓	–	^263^
	TFEB	Rat NPCs (TBHP)	ROS↓ apoptosis↓ autophagy↓ Senescence↓	–	^264^
	MiR‐4478	Human NPCs (H_2_O_2_)	ROS↓ apoptosis↓	–	^265^

### 7.2 ROS scavenger

Mitoquone (MitoQ), a mitochondria‐targeted antioxidant, contains lipophilic triphenylphosphonium cation and coenzyme Q10, which accumulate in large quantities on the inner mitochondrial membrane surface and have the function of ROS scavenging and reducing mitochondrial oxidative damage.^238^ Currently, MitoQ has been widely used to treat various diseases, including cardiovascular diseases, neurodegenerative diseases, and ischemia–reperfusion injury.^239^, ^240^ Kang et al.^25^ reported that MitoQ treatment inhibited compression‐induced oxidative stress and apoptosis in human NPCs and validated its efficacy in an in vitro compression model of IVD. In terms of improving mitochondrial function, MitoQ treatment significantly reduced the production of ROS in the mitochondria of NPCs, ameliorated the abnormal opening of mPTP and the reduction of *ΔΨ*m, and restored the compression induced imbalance between mitochondrial fission and fusion in human NPCs. In addition, MitoQ promotes mitochondrial autophagy by activating PINK1/Parkin signal, restoring lysosomal protease activity and low PH environment, and improving the disturbance of autophagosome and lysosome fusion. Further mechanism studies have shown that the therapeutic effect of MitoQ is mediated by Nrf2 signal pathway. GSH, a major antioxidant for maintaining cellular redox homeostasis, can inhibit apoptosis and ECM degradation in human NPCs induced by H_2_O_2_.^241^ In addition, N‐acetylcysteine (NAC), a GSH precursor, can reduce H_2_O_2_‐induced apoptosis and CEP calcification by down‐regulating the ROS/MAPK/NF‐κB signalling pathway.^143^

### 7.3 Hormone

Melatonin (MT), an indole hormone released by the pineal gland, has a wide range of physiological functions such as physiological rhythm regulation, anti‐inflammatory, anti‐aging and anti‐oxidative stress, and has been intensively studied in tumours, psychosocial disorders, neurodegenerative diseases and osteoarticular diseases.^130^, ^242^ In IDD, MT effectively inhibited oxidative stress‐induced apoptosis in NPCs and maintained ECM homeostasis by upregulating the expression of Col II, Agg and Sox‐9 and downregulating the expression of MMP‐13 and ADAMTS‐5.^90^, ^176^ In addition, autophagy impairment is an important pathogenic factor in oxidative stress‐induced IDD. MT can promote the expression of LC3II/I and beclin‐1 to induce autophagic phenotype by activating the PINK1/parkin signalling axis.^176^ Estradiol (E2), the main female hormone, is important for the regulation of bone homeostasis, and its deficiency is one of the main causes of osteoporosis in postmenopausal women.^243^ In the IDD study, the prevalence and severity of IDD in postmenopausal women were significantly higher than those in men of the same age, which also existed in ovariectomized rats.^244^, ^245^, ^246^ Further studies showed that the levels of serum total antioxidant capacity (T‐AOC), SOD, GSHPx and GSH in E2 treated rats were significantly higher than those in ovariectomized rats, suggesting that E2 may delay IDD by restoring the balance of redox in IVD.^52^ In addition, parathyroid hormone (PTH) is one of the essential hormones for bone remodelling and maintenance of calcium homeostasis. PTH can inhibit inflammation, oxidative stress, apoptosis and ECM degradation through the CAMP response element binding protein (CREB)/Sonic hedgehog (SHH) signalling axis to improve the survival and function of NPCs.^247^ Recently, Zhao et al.^22^ showed that cortistatin (CST) expression level was negatively correlated with Pfirrmann grade. the expression level of CST in NP tissues of 10‐month‐old mice was significantly lower than that of 2‐month‐old mice. Importantly, CST knockout mice showed oxidative stress, catabolism and apoptosis phenotypes. In vitro, CST treatment attenuated IL‐1β‐and TNF‐α‐induced oxidative stress, catabolism and apoptosis in NPCs by inhibiting the NF‐κB/NLRP3 signalling axis.

### 7.4 Medicine

Vitamin D is a commonly used drug in the treatment of osteoporosis, which can affect cell proliferation, differentiation, apoptosis and redox balance.^248^ With the increase of the degree of IVD degeneration, the expression of vitamin D receptor (VDR) decreased, while exogenous 1α,25‐dihydroxyvitamin D_3_ (1,25(OH)_2_D_3_) could effectively delay the progression of IDD.^141^, ^171^ In vitro, 1,25(OH)_2_D_3_ treatment effectively inhibited oxidative stress, senescence, apoptosis and ECM degradation in IVD cells caused by H_2_O_2_ and IL‐1β through upregulation of PI3K/Akt signalling pathway and downregulation of NF‐κB signalling pathway.^121^, ^141^ In addition, 1,25(OH)_2_D_3_ attenuates excessive autophagy and inhibits H_2_O_2_‐induced apoptosis by upregulating the mTOR/p70S6K signalling pathway.^171^ In addition to vitamin D, diabetes therapeutics have also been found to have unique effects in delaying IDD. Metformin (MF) treatment inhibits TBHP‐induced apoptosis and ECM degradation in NPCs and suppresses cellular senescence by downregulating the p53/p21/Rb and p16/Rb signalling axes.^249^ In addition, MF can promote cell autophagy and alleviate oxidative stress injury by regulating AMPK and cGAS‐STING signalling pathways.^208^, ^249^ Recently, Liu et al.^250^ showed that pioglitazone (PGZ) could inhibit compression induced oxidative stress in nucleus pulposus mesenchymal stem cells (NPMSCs) and attenuate mitochondrial dysfunction and apoptosis. In addition, aspirin, ulinastatin, and isopentobarbital have also been reported to delay IDD progression by inhibiting oxidative stress.^148^, ^251^, ^252^

### 7.5 Biomaterial

At present, biomaterials based on tissue engineering strategies have been proved to have the functions of regulating local microenvironment homeostasis, maintaining IVD phenotype and promoting IVD repair in preclinical models.^253^ Prussian blue nanoparticles (PBNPs) have excellent biocompatibility and unique antioxidant properties, which have been extensively studied in biomedical engineering.^254^ In terms of antioxidation, PBNPs can increase the activities of GSH and SOD, GPX and glutathione reductase (GR) in NPCs under the action of H_2_O_2_. More importantly, PBNPs can inhibit the ubiquitination modification of SOD1, thereby inhibiting the degradation of the enzyme through the proteasome pathway. In addition, PBNPs can ameliorate H_2_O_2_‐induced proliferation and inflammatory response of NPCs by regulating the expression of PCNA, p53 and inflammatory factor interferon β1 (Ifnβ1). In terms of regulating ECM metabolism, PBNPs maintained ECM quality by down‐regulating the expression of MMP‐3, 9, 13 and ADAMTS‐5 and up‐regulating the expression of Col II a1 and Agg.^255^ Recently, Yang et al.^256^ designed a special injectable hydrogel PBNPs@OBG by double dynamic bond cross‐linking between oxidized hyaluronic acid (OHA), borax and gelatin. PBNPs@OBG has the properties of injectability, repeatability, self‐healing, adhesion, antibacterial and long‐term retention, which can provide stable and sustainable release of PBNPs. In addition, Bai et al.^257^ developed a ROS scavenging stent (Rapa@Gel) loaded with rapamycin, which has the function of scavenging ROS and continuously releasing rapamycin. Within the acupuncture‐induced degenerative IVD in rats, Rapa@Gel could effectively inhibit ROS levels and ECM degradation and promote regeneration of IVD in rats. Interestingly, rapa@Gel could also reduce the inflammatory response by inducing macrophage M2 polarization.

### 7.6 Cell therapy

In addition to the above approaches, stem cell‐based therapies are providing increasing evidence in the repair and regeneration of IVD. Studies have shown that the antioxidant effect mediated by exosomes (exos) secreted from stem cells is one of the reasons for delaying IDD.^258^ Mesenchymal stem cell (MSC)‐derived exos treatment effectively attenuates H_2_O_2_‐induced apoptosis and inflammatory response in NPCs^259^ In addition, CEP degeneration is an important factor contributing to the development and progression of IDD. Lin et al.^260^ showed that miroRNA (miR)‐31‐5p in MSC exos significantly alleviates TBHP‐induced apoptosis and calcification of CEP cells by negatively regulating the activating transcription factor 6 (ATF6) related ER stress. In addition, Luo et al.^261^ found that compared with degenerative CEP stem cell‐derived exosomes (D‐exos), normal CEP stem cell‐derived exosomes (N‐exos) can inhibit TBHP‐induced NPCs apoptosis and delay the progression of IDD by activating autophagy mediated by PI3K/AKT pathway.

### 7.7 Others

Normal IVD cells are in a hypoxic microenvironment, in which hypoxia stress can activate hypoxia inducible factor‐1α (HIF‐1α)‐mediated hypoxia response element (HRE)‐dependent gene transcription to maintain IVD cell survival and metabolism.^262^ In vitro, HIF‐1α can inhibit inflammation, metabolic disorder and apoptosis of NPCs in mice by alleviating mitochondrial dysfunction.^263^ In addition, TFEB acts as a member of the leucinezipper family, which promotes autophagy by inducing lysosome biogenesis and autophagosome formation.^139^ Studies have shown that overexpression of TFEB can inhibit TBHP‐induced apoptosis and senescence of NPCs by restoring autophagy flux, and reduce puncture‐induced IDD.^264^ In addition, lncRNAs are also involved in the regulation of oxidative stress in IDD, which provides a new therapeutic target for IDD.^265^

## 8 CONCLUSION AND PROSPECT

IDD is the main cause of LBP and disability, especially in an aging society. Therefore, it is urgent to further elucidate the pathogenesis of IDD and develop targeted therapeutic approaches. In recent years, increasing evidence has supported the important role of oxidative stress in the pathophysiology of IDD, and these potential effects alter IVD homeostasis and contribute to the development of IDD. The effect of oxidative stress on IVD degeneration is multi‐level and complex. On the one hand, oxidative stress reduces the number of normal IVD cells by activating the internal death mechanism of IVD cells and senescence‐related genes. On the other hand, ROS and pro‐inflammatory cytokines change the microenvironment of IVD cells, resulting in the imbalance between anabolism and catabolism, which reduces the content of extracellular matrix in IVD. The synergistic effect of the two promotes the abnormal change of the normal organizational structure of IVD, which eventually leads to the degeneration of IVD. However, the intrinsic molecular mechanism by which oxidative stress promotes IDD progression has not been fully elucidated and needs to be continuously explored by more investigators. In combination with current preclinical studies, numerous antioxidant strategies have shown excellent effects for the treatment of IDD, but key clinical evidence is still lacking to clarify the exact efficacy of antioxidants. In this review, we mainly summarize the potential relationship between IDD and oxidative stress, including the disorder of redox state in degenerative IVD, the oxidative damage of ROS to biological macromolecules, the signal pathways related to oxidative stress and the role of oxidative stress in IVD cell death, aging, autophagy, ECM metabolism and inflammation. In addition, we review the progress of research on antioxidant therapy in IDD. Future research may focus on the direct mechanism of oxidative stress promoting the progress of IDD, which is crucial for further elucidating the pathogenesis and developing targeted therapies.

## AUTHOR CONTRIBUTIONS

Yidian Wang, Huiguang Chen, and Tao Wang contributed equally to this work and were listed as a co‐first author. All authors contributed to the revision and approved the submitted version.

## CONFLICT OF INTEREST

The authors declare no conflict of interest.

## Data Availability

Data sharing not applicable to this article as no data sets were generated or analysed during the current study.
